# Supplementary material for: Evaluating and integrating spatial capture–recapture models with data of variable individual identifiability
Source: Ecol Appl. 2021 Aug 11;31(7):e02405. doi: 10.1002/eap.2405 (PMC9286611; doi:10.1002/eap.2405)
Supplement: Supplementary file 1 — Appendix S1 [file EAP-31-0-s002.pdf]

Supporting Information. Ruprecht, J.S., C.E. Eriksson, T.D. Forrester, D.A. Clark, M.J. Wisdom, M.M. Rowland, B.K. Johnson, and T. Levi. 2021. Evaluating and integrating spatial capture–recapture models with data of variable individual identifiability. Ecological Applications.

Appendix S1: Literature review of density estimation methods for carnivores, 2015–2020.

We queried Google Scholar between 18 and 20 March 2020 to attain a sample of the methods used to estimate carnivore population densities using search terms "carnivore density estimation" and "carnivore abundance estimation." For each search term we reviewed the first 250 records only. We only utilized studies that estimated the absolute population density or abundance (i.e. not relative measures or indices of abundance or density) of terrestrial carnivores between 2015 and 2020. The methods in each study were classified into one of nine categories: spatial count (SC; Chandler and Royle 2013), spatial mark-resight (SMR; Sollmann et al. 2013), spatial capture-recapture (SCR; Royle et al. 2013), capture-recapture (e.g. Otis et al. 1978, Nichols 1992), mark-resight (e.g. White 1996, McClintock and White 2009), Royle-Nichols model (Royle and Nichols 2003), random encounter model (Rowcliffe et al. 2008), N-mixture model (Royle 2004), distance sampling (Buckland et al. 1993), and other.

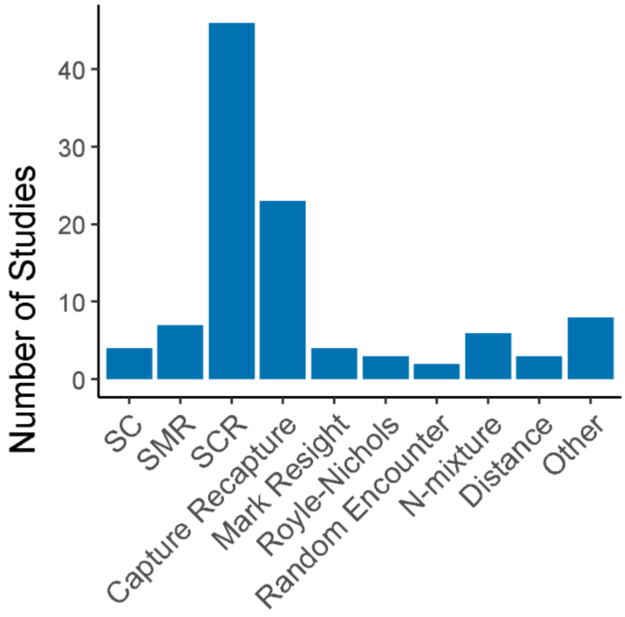

Figure S1: The frequency of methods used for the density estimation of terrestrial carnivores between 2015 and 2020 (N = 87 studies reporting methods 106 times).

Literature cited

Buckland, S. T., D. R. Anderson, K. P. Burnham, and J. L. Laake. 1993. Distance sampling: estimating abundance of biological populations. Oxford University Press, Oxford, UK.

Chandler, R. B., and J. A. Royle. 2013. Spatially explicit models for inference about density in unmarked or partially marked populations. The Annals of Applied Statistics 7:936–954.

McClintock, B. T., and G. C. White. 2009. A less field-intensive robust design for estimating demographic parameters with mark–resight data. Ecology 90:313–320.

Nichols, J.D. 1992. Capture-recapture models. BioScience 42:94–102.

Otis, D. L., K. P. Burnham, G. C. White, and D. R. Anderson. 1978. Statistical inference from capture data on closed animal populations. Wildlife Monographs 62:3–135.

Rowcliffe, J. M., J. Field, S. T. Turvey, and C. Carbone. 2008. Estimating animal density using camera traps without the need for individual recognition. Journal of Applied Ecology 45:1228–1236.

Royle, J. A. 2004. N-mixture models for estimating population size from spatially replicated counts. Biometrics 60:108–115.

Royle, J. A., and J. D. Nichols. 2003. Estimating abundance from repeated presence–absence data or point counts. Ecology 84:777–790.

Royle, J. A., R. B. Chandler, R. Sollmann, and B. Gardner. 2013. Spatial capture-recapture. Academic Press.

Sollmann, R., B. Gardner, A. W. Parsons, J. J. Stocking, B. T. McClintock, T. R. Simons, K. H. Pollock, and A. F. O'Connell. 2013. A spatial mark–resight model augmented with telemetry data. Ecology 94:553–559.

White, G. C. 1996. NOREMARK: population estimation from mark-resighting surveys. Wildlife Society Bulletin 24:50–52.
