# Supplementary material for: Evaluating and integrating spatial capture–recapture models with data of variable individual identifiability
Source: Ecol Appl. 2021 Aug 11;31(7):e02405. doi: 10.1002/eap.2405 (PMC9286611; doi:10.1002/eap.2405)
Supplement: Supplementary file 4 — Appendix S4 [file EAP-31-0-s008.pdf]

Supporting Information. Ruprecht, J.S., C.E. Eriksson, T.D. Forrester, D.A. Clark, M.J. Wisdom, M.M. Rowland, B.K. Johnson, and T. Levi. 2021. Evaluating and integrating spatial capture–recapture models with data of variable individual identifiability. Ecological Applications.

Appendix S4: Tables including measures of central tendency, detection parameters, credible intervals, and coefficients of variation for base density estimation models for black bears, bobcats, cougars, and coyotes. Models include spatial count (SC), conventional spatial mark-resight (SMR), generalized spatial mark-resight (gSMR), spatial capture-recapture (SCR), and a novel hybrid model combining SCR and gSMR. Each model is fit with and without global positioning system (GPS) collar data from a subset of individuals within each population. See Appendix S6 for similar tables for sex-specific models, Appendix S7 for models with reduced sampling periods, and Appendix S8 for models incorporating behavioral status of coyotes.

Table S1: Density estimates for black bears across a suite of models. Densities are presented as the number of animals per 100 km^2. HPDI = highest posterior density interval. BCI = Bayesian Credible Interval. CV = coefficient of variation (defined as standard deviation divided by the posterior mean).

**Model**	**Mean**	**Median**	**Mode**	**SD**	**Lower 95% HPDI**	**Upper 95% HPDI**	**Lower 95% BCI**	**Upper 95% BCI**	**CV**
SC	72.83	70.06	61.49	22.27	38.69	115.59	36.8	114.65	0.31
SC + GPS	0.99	0.74	0.45	0.92	0.12	2.65	0.2	3.36	0.93
SPA	85.41	82.26	76.1	30.65	37.85	144.5	33.12	141.95	0.36
SPA + GPS	0.91	0.62	0.43	0.95	0.08	2.58	0.2	3.28	1.04
SMR	7.66	7.53	7.08	2.85	2.61	13.19	2.34	12.95	0.37
SMR + GPS	12.49	12.21	11.93	2.94	7.26	18.69	7.61	19.43	0.24
gSMR	7.9	7.88	7.83	2.89	2.85	13.38	2.34	13.11	0.37
gSMR + GPS	11.1	10.93	10.75	2.47	6.75	16.23	6.83	16.47	0.22
SCR	11.17	10.86	9.65	4.06	5.08	19.21	4.51	18.71	0.36
SCR + GPS	10.31	10.2	9.9	2.46	6.2	15.06	6.05	14.94	0.24
SCR + gSMR	12.27	12.13	11.74	2.8	7.28	17.71	7.32	17.83	0.23
SCR + gSMR + GPS	8.3	8.12	7.93	1.48	5.78	11.44	5.85	11.71	0.18

Table S2: Detection parameters for models estimating the density of black bears. Values are presented as the median of the posterior distribution with 95% Bayesian Credible Intervals in parentheses. σ = spatial scale parameter, $\lambda0_{resight}$ = baseline detection rate for camera analyses, $\lambda0_{marking}$ = baseline detection rate for the marking process, $p0$ (intercept) = baseline detection probability on the logit scale for genetic SCR models when all covariates are at zero, $p0$ (survey effort) = effect of survey effort (distance traveled by scat detection dogs) on baseline detection rate in genetic SCR models on the logit scale.

Model	σ	$\lambda0_{resight}$	$\lambda0_{marking}$	$p0$ (intercept)	$p0$ (survey effort)
SC	294.34 (213.37–409.32)	0.5 (0.32–0.78)			
SC + GPS	6484.6 (6218.35–6795.88)	0.15 (0.02–1.08)			
SPA	345.17 (233.29–562.33)	0.22 (0.12–0.36)			
SPA + GPS	6496.33 (6215.94–6791.69)	0.13 (0.02–0.61)			
SMR	4133.23 (3007.07–6587.71)	0.03 (0.01–0.05)			
SMR + GPS	3395.24 (3243.42–3541.84)	0.02 (0.01–0.03)			
gSMR	3762.05 (2790.67–5921.49)	0.03 (0.02–0.06)	0.09 (0.03–0.23)		
gSMR + GPS	3387.32 (3248.05–3543.1)	0.03 (0.02–0.04)	0.07 (0.03–0.15)		
SCR	6111.9 (3497.58–9723.03)			-5.07 (-5.92–-4.21)	0.43 (0.17–0.66)
SCR + GPS	6487.4 (6225.27-6786.06)			-5.2 (-5.77–-4.6)	0.42 (0.15–0.66)
gSMR + SCR	3736.73 (2979.98–5117)	0.02 (0.01–0.03)	0.05 (0.02–0.1)	-4.26 (-4.79–-3.75)	0.4 (0.13–0.65)
gSMR + SCR + GPS	5269.59 (5046.36–5506.09)	0.01 (0.01–0.02)	0.04 (0.02–0.08)	-4.53 (-5.01–-4.09)	0.41 (0.14–0.65)

Table S3: Density estimates for bobcats across a suite of models. Densities are presented as the number of animals per 100 km^2. HPDI = highest posterior density interval. BCI = Bayesian Credible Interval. CV = coefficient of variation (defined as standard deviation divided by the posterior mean).

**Model**	**Mean**	**Median**	**Mode**	**SD**	**Lower 95% HPDI**	**Upper 95% HPDI**	**Lower 95% BCI**	**Upper 95% BCI**	**CV**
SC	103.23	92.86	37.65	62.57	12.26	215.88	18.85	224.37	0.61
SC + GPS	7.53	5.81	3.85	5.39	0.82	20.31	1.31	21.79	0.72
SPA	86.03	82.49	66.77	38.47	23.57	156.02	25.45	157.91	0.45
SPA + GPS	17.26	6.13	2.44	29.58	1.41	83.9	1.41	131.53	1.71
SMR	9.54	8.44	7.23	4.78	2.62	20.07	3.03	20.64	0.5
SMR + GPS	8.06	7.37	6.15	3.49	2.13	15.48	2.87	17.28	0.43
gSMR	8.45	8.03	7.16	3.7	1.72	14.99	2.62	16.38	0.44
gSMR + GPS	8.1	7.37	6.72	3.46	2.54	14.82	3.36	17.44	0.43
SCR	13.29	12.94	12.14	2.55	8.6	18.02	9.25	18.84	0.19
SCR + GPS	12.03	11.96	12.1	2.03	8.27	15.97	8.44	16.22	0.17
SCR + gSMR	12.33	12.12	11.87	2.1	8.44	16.46	8.68	16.87	0.17
SCR + gSMR + GPS	11.75	11.63	11.45	1.73	8.52	14.99	8.76	15.32	0.15

Table S4: Detection parameters for models estimating the density of bobcats. Values are presented as the median of the posterior distribution with 95% Bayesian Credible Intervals in parentheses. σ = spatial scale parameter, $\lambda0_{resight}$ = baseline detection rate for camera analyses, $\lambda0_{marking}$ = baseline detection rate for the marking process, $p0$ (intercept) = baseline detection probability on the logit scale for genetic SCR models when all covariates are at zero, $p0$ (survey effort) = effect of survey effort (distance traveled by scat detection dogs) on baseline detection rate in genetic SCR models on the logit scale.

**Model**	**σ**	$\boldsymbol{\lambda0_{resight}}$	$\boldsymbol{\lambda0_{marking}}$	$\boldsymbol{p0}$ **(intercept)**	$\boldsymbol{p0}$ **(survey effort)**
SC	166.48 (84.39–409.88)	0.19 (0.06–0.5)			
SC + GPS	1829.55 (1736.68–1940.11)	0.03 (0.01–0.09)			
SPA	186.61 (107.6–350.55)	0.21 (0.08–0.46)			
SPA + GPS	1827.85 (1723.6–1934.68)	0.03 (0–0.11)			
SMR	1211.64 (761.43–2282.95)	0.05 (0.02–0.11)			
SMR + GPS	1831.78 (1734.15–1941.38)	0.03 (0.01–0.05)			
gSMR	1267.78 (829.44–2366.31)	0.05 (0.02–0.11)	0.37 (0.07–1.68)		
gSMR + GPS	1826.63 (1731.43–1928.51)	0.03 (0.01–0.05)	0.11 (0.03–0.29)		
SCR	1645.31 (1373.42–2067.29)			-1.99 (-2.55–-1.47)	0.44 (0.19–0.75)
SCR + GPS	1822.17 (1724.77–1928.05)			-2.15 (-2.58–-1.78)	0.44 (0.2–0.71)
gSMR + SCR	1603.13 (1372.31–1887.32)	0.02 (0.01–0.04)	0.13 (0.03–0.33)	-1.91 (-2.38–-1.42)	0.46 (0.19–0.77)
gSMR + SCR + GPS	1816.29 (1728.16–1917.57)	0.02 (0.01–0.03)	0.09 (0.02–0.2)	-2.13 (-2.51–-1.78)	0.44 (0.19–0.72)

Table S5: Density estimates for cougars across a suite of models. Densities are presented as the number of animals per 100 km^2. HPDI = highest posterior density interval. BCI = Bayesian Credible Interval. CV = coefficient of variation (defined as standard deviation divided by the posterior mean).

**Model**	**Mean**	**Median**	**Mode**	**SD**	**Lower 95% HPDI**	**Upper 95% HPDI**	**Lower 95% BCI**	**Upper 95% BCI**	**CV**
SC	46.07	44.39	37.32	13.81	25.68	74.69	23.02	72.62	0.3
SC + GPS	1.14	0.66	0.31	1.31	0.08	4.21	0.12	5.35	1.14
SPA	101.53	99.71	70.36	49.5	25.27	190.2	23.89	189.03	0.49
SPA + GPS	3	1.99	0.52	2.65	0.08	8.39	0.2	9.01	0.88
SMR	2.36	2.26	2.04	1.08	0.27	4.37	0.59	4.88	0.46
SMR + GPS	1.76	1.72	1.71	0.57	0.74	2.89	0.82	3.08	0.33
gSMR	2.37	2.26	2.14	0.84	0.86	3.94	1.09	4.49	0.36
gSMR + GPS	1.71	1.68	1.61	0.5	0.82	2.69	0.86	2.77	0.29
SCR	1.51	1.26	0.44	1.05	0.23	3.64	0.3	4.24	0.69
SCR + GPS	2	1.89	1.73	0.7	0.66	3.34	0.89	3.64	0.35
SCR + gSMR	2.25	2.15	2.11	0.78	0.96	3.74	1.13	4.17	0.35
SCR + gSMR + GPS	1.92	1.85	1.75	0.55	0.89	2.98	1.03	3.18	0.29

Table S6: Detection parameters for models estimating the density of cougars. Values are presented as the median of the posterior distribution with 95% Bayesian Credible Intervals in parentheses. σ = spatial scale parameter, $\lambda0_{resight}$ = baseline detection rate for camera analyses, $\lambda0_{marking}$ = baseline detection rate for the marking process, $p0$ (intercept) = baseline detection probability on the logit scale for genetic SCR models when all covariates are at zero, $p0$ (survey effort) = effect of survey effort (distance traveled by scat detection dogs) on baseline detection rate in genetic SCR models on the logit scale.

Model	σ	$\lambda0_{resight}$	$\lambda0_{marking}$	$p0$ (intercept)	$p0$ (survey effort)
SC	323.16 (211.81–540.19)	0.19 (0.08–0.45)			
SC + GPS	5064.59 (4861.19–5263.66)	0.09 (0.01–0.7)			
SPA	261.47 (143.15–571.68)	0.14 (0.04–0.3)			
SPA + GPS	5071.66 (4868.53–5287.98)	0.02 (0–0.26)			
SMR	3390.68 (2454.34–6989.67)	0.03 (0.02–0.06)			
SMR + GPS	5040.82 (4847.15–5248.14)	0.02 (0.01–0.03)			
gSMR	3376.29 (2521.17–4930.78)	0.04 (0.02–0.06)	0.43 (0.15–1.08)		
gSMR + GPS	5188.22 (4993.8–5395.69)	0.02 (0.01–0.03)	0.3 (0.11–0.72)		
SCR	5094.4 (2646.07–14258.39)			-3.67 (-5.12–0.66)	0.58 (0.1–8.72)
SCR + GPS	4276.98 (4119.95–4454.28)			-3.75 (-4.71–-2.89)	0.53 (0.05–0.96)
gSMR + SCR	3794.68 (2860.59–5377.93)	0.03 (0.02–0.05)	0.4 (0.14–0.95)	-3.57 (-4.36–-2.81)	0.54 (0.07–0.99)
gSMR + SCR + GPS	4427.46 (4241.86–4612.95)	0.03 (0.02–0.04)	0.42 (0.15–1.01)	-3.67 (-4.45–-3.01)	0.52 (0.05–0.97)

Table S7: Density estimates for coyotes across a suite of models. Densities are presented as the number of animals per 100 km^2. HPDI = highest posterior density interval. BCI = Bayesian Credible Interval. CV = coefficient of variation (defined as standard deviation divided by the posterior mean).

**Model**	**Mean**	**Median**	**Mode**	**SD**	**Lower 95% HPDI**	**Upper 95% HPDI**	**Lower 95% BCI**	**Upper 95% BCI**	**CV**
SC	107.87	107.57	106.93	21.02	70.49	146.94	66.07	144.65	0.19
SC + GPS	2.55	2.29	2.04	1.04	0.76	4.58	1.07	5.19	0.41
SPA	145.69	146.59	147.17	49.03	66.46	235.68	54.68	229.55	0.34
SPA + GPS	7.76	6.6	5.01	4.57	2.36	16.5	2.36	20.27	0.59
SMR	45.88	45.47	44.99	7.29	31.13	59.35	31.89	60.27	0.16
SMR + GPS	33.23	32.96	32.36	5.16	23.8	43.64	24.26	44.86	0.16
gSMR	41.29	40.89	40.06	5.88	30.06	52.34	30.67	53.41	0.14
gSMR + GPS	31.95	31.74	31.45	4.23	23.65	39.98	24.26	40.59	0.13
SCR	33.42	33.24	32.79	3.39	27.17	40.28	27.31	40.69	0.1
SCR + GPS	25.38	25.24	25.02	2.58	20.83	30.48	20.97	30.9	0.1
SCR + gSMR	36.87	36.55	36.06	3.58	30.48	44.41	30.21	44.28	0.1
SCR + gSMR + GPS	27.63	27.59	27.52	2.46	23.03	32.41	23.03	32.55	0.09

Table S8: Detection parameters for models estimating the density of coyotes. Values are presented as the median of the posterior distribution with 95% Bayesian Credible Intervals in parentheses. σ = spatial scale parameter, $\lambda0_{resight}$ = baseline detection rate for camera analyses, $\lambda0_{marking}$ = baseline detection rate for the marking process, $p0$ (intercept) = baseline detection probability on the logit scale for genetic SCR models when all covariates are at zero, $p0$ (survey effort) = effect of survey effort (distance traveled by scat detection dogs) on baseline detection rate in genetic SCR models on the logit scale.

Model	σ	$\lambda0_{resight}$	$\lambda0_{marking}$	$p0$ (intercept)	$p0$ (survey effort)
SC	315.11 (256.58–394.84)	0.87 (0.63–1.16)			
SC + GPS	2670.98 (2586.77–2762.85)	0.79 (0.36–1.15)			
SPA	345.32 (249.37–518.03)	0.39 (0.26–0.55)			
SPA + GPS	2727.25 (2641.34–2818.4)	0.19 (0.06–0.45)			
SMR	636.71 (581.58–698.39)	0.56 (0.43–0.74)			
SMR + GPS	2626.96 (2545.21–2709.74)	0.05 (0.03–0.06)			
gSMR	779.08 (714.49–886.1)	0.41 (0.29–0.55)	0.16 (0.07–0.3)		
gSMR + GPS	2644.01 (2563.03–2727.6)	0.05 (0.04–0.06)	0.02 (0.01–0.03)		
SCR	1288.67 (1162.51–1472.42)			-1.43 (-1.8–-1.06)	0.59 (0.36–0.89)
SCR + GPS	2606.66 (2526.14–2692.74)			-2.69 (-2.95–-2.43)	0.4 (0.25–0.56)
gSMR + SCR	1188.66 (1094.8–1315.22)	0.18 (0.15–0.22)	0.07 (0.03–0.12)	-1.33 (-1.63–-1.05)	0.6 (0.37–0.89)
gSMR + SCR + GPS	2582.49 (2507.24–2666.19)	0.05 (0.05–0.06)	0.02 (0.01–0.04)	-2.75 (-2.97–-2.52)	0.38 (0.24–0.53)
