# Supplementary material for: Evaluating and integrating spatial capture–recapture models with data of variable individual identifiability
Source: Ecol Appl. 2021 Aug 11;31(7):e02405. doi: 10.1002/eap.2405 (PMC9286611; doi:10.1002/eap.2405)
Supplement: Supplementary file 6 — Appendix S6 [file EAP-31-0-s005.pdf]

Supporting Information. Ruprecht, J.S., C.E. Eriksson, T.D. Forrester, D.A. Clark, M.J. Wisdom, M.M. Rowland, B.K. Johnson, and T. Levi. 2021. Evaluating and integrating spatial capture–recapture models with data of variable individual identifiability. Ecological Applications.

Appendix S6: Tables including measures of central tendency, detection parameters, credible intervals, and coefficients of variation for sex-specific density estimation models for black bears, bobcats, cougars, and coyotes. Models include spatial count (SC), spatial mark resight (SMR), generalized spatial mark resight (gSMR), spatial capture recapture (SCR), and a novel hybrid model combining SCR and gSMR. Each model is fit with and without global positioning system (GPS) collar data from a subset of individuals within each population. See Appendix S4 for similar tables for base models, Appendix S7 for models with reduced sampling periods, and Appendix S8 for models incorporating behavioral status of coyotes.

Table S1: Sex-specific density estimates for black bears across a suite of models. Densities are presented as the number of animals per 100 km^2. HPDI = highest posterior density interval. BCI = Bayesian Credible Interval. CV = coefficient of variation (defined as standard deviation divided by the posterior mean).

**Model**	**Mean**	**Median**	**Mode**	**SD**	**Lower 95% HPDI**	**Upper 95% HPDI**	**Lower 95% BCI**	**Upper 95% BCI**	**CV**
SMR	17.94	17.72	17.3	4.27	10.77	26.65	9.95	26.03	0.24
SMR + GPS	12.72	12.17	11.48	3.46	6.83	19.9	7.3	21.07	0.27
gSMR	21.32	21.09	20.72	5.33	11.15	31.22	11.88	32.55	0.25
gSMR + GPS	11.8	11.45	11.03	3.18	6.13	18.14	6.75	19.16	0.27
SCR	13.95	14.25	15.71	5.91	3.54	23.99	3.43	23.91	0.42
SCR + GPS	11.07	11.17	11.63	2.41	6.89	15.33	6.43	15.09	0.22
SCR + gSMR	19.72	19.59	18.81	4.33	11.28	27.76	11.43	27.96	0.22
SCR + gSMR + GPS	9.22	9.05	8.93	1.79	6.04	12.77	6.28	13.31	0.19

Table S2: Detection parameters for sex-specific models estimating the density of black bears. Values are presented as the median of the posterior distribution with 95% Bayesian Credible Intervals in parentheses. σ = spatial scale parameter, $\lambda 0_{resight}$ = baseline detection rate for camera analyses, $\lambda 0_{marking}$ = baseline detection rate for the marking process, $p0$ (intercept) = baseline detection probability on the logit scale for genetic SCR models when all covariates are at zero, $p0$ (survey effort) = effect of survey effort (distance traveled by scat detection dogs) on baseline detection probability in genetic SCR models on the logit scale, and $p0$(sex) = effect of being male (compared to females) on baseline detection probability in genetic SCR models on the logit scale.

Model	σ female	σ male	$\lambda 0_{resight}$ female	$\lambda 0_{resight}$ male	$\lambda 0_{marking}$	$p0$ (intercept)	$p0$ (survey effort)	$p0$ (sex)
SMR	560.89 (469.37–691.29)	5269.79 (3289.43–12821.16)	0.41 (0.24–0.62)	0.02 (0.01–0.06)				
SMR + GPS	1953.52 (1828.38–2096.05)	4084.05 (3863.52–4324.39)	0.06 (0.03–0.12)	0.02 (0.01–0.03)				
gSMR	574.28 (481.44–696.47)	5550.45 (3340.04–9767.54)	0.36 (0.22–0.54)	0.03 (0.01–0.09)	0.27 (0.09–0.77)			
gSMR + GPS	1956.63 (1829.93–2097.87)	4077.95 (3864.93–4323.61)	0.07 (0.03–0.13)	0.02 (0.01–0.03)	0.09 (0.03–0.2)			
SCR	1850.09 (706.43–18278.82)	7461.1 (3786.02–18730.44)				-3.65 (-6.64–-1.42)	0.43 (0.16–0.68)	-1.71 (-4.3–1.41)
SCR + GPS	4475.59 (4190.24–4795)	7537.76 (7130.07–7981.75)				-4.81 (-5.56–-4.04)	0.42 (0.17–0.65)	-0.5 (-1.56–0.51)
gSMR + SCR	827.36 (618.04–1261.23)	5386.28 (3679.99–8982)	0.14 (0.05–0.27)	0.01 (0.01–0.02)	0.07 (0.03–0.16)	-2.28 (-3.26–-1.38)	0.44 (0.15–0.71)	-2.04 (-3.15–-0.96)
gSMR + SCR + GPS	3647.2 (3406.83–3914.74)	6136.59 (5813.99–6494.81)	0.02 (0.01–0.04)	0.01 (0.01–0.02)	0.04 (0.02–0.08)	-4.24 (-5–-3.58)	0.41 (0.14–0.64)	-0.36 (-1.24–0.55)

Table S3: Sex-specific density estimates for bobcats across a suite of models. Densities are presented as the number of animals per 100 km^2. HPDI = highest posterior density interval. BCI = Bayesian Credible Interval. CV = coefficient of variation (defined as the standard deviation divided by the posterior mean).

**Model**	**Mean**	**Median**	**Mode**	**SD**	**Lower 95% HPDI**	**Upper 95% HPDI**	**Lower 95% BCI**	**Upper 95% BCI**	**CV**
SMR	10.77	9.83	7.49	6.32	0.57	22.6	1.23	23.51	0.59
SMR + GPS	11.53	10.73	8.47	5.31	3.19	22.44	3.52	23.01	0.46
gSMR	10.16	9.75	8.92	5.72	0.33	20.39	0.82	22.03	0.56
gSMR + GPS	10.43	9.58	8.61	4.5	2.87	19.66	3.77	21.13	0.43
SCR	13.73	13.43	12.94	2.85	8.35	19.33	9.09	20.31	0.21
SCR + GPS	12.96	12.78	12.38	2.39	8.85	17.85	9.01	18.18	0.18
SCR + gSMR	12.61	12.53	12.27	2.07	8.85	16.71	8.93	16.95	0.16
SCR + gSMR + GPS	11.59	11.47	11.23	1.88	8.27	15.32	8.35	15.48	0.16

Table S4: Detection parameters for sex-specific models estimating the densities of bobcats. Values are presented as the median of the posterior distribution with 95% Bayesian Credible Intervals in parentheses. σ = spatial scale parameter, $\lambda 0_{resight}$ = baseline detection rate for camera analyses, $\lambda 0_{marking}$ = baseline detection rate for the marking process, $p0$ (intercept) = baseline detection probability on the logit scale for genetic SCR models when all covariates are at zero, $p0$ (survey effort) = effect of survey effort (distance traveled by scat detection dogs) on baseline detection probability in genetic SCR models on the logit scale, and $p0$(sex) = effect of being male (compared to females) on baseline detection probability in genetic SCR models on the logit scale.

Model	σ female	σ male	$\lambda 0_{resight}$ female	$\lambda 0_{resight}$ male	$\lambda 0_{marking}$	$p0$ (intercept)	$p0$ (survey effort)	$p0$ (sex)
SMR	2166.68 (663.6–15779.53)	1048.94 (681.65–4895.78)	0.01 (0–0.08)	0.16 (0.04–2.32)				
SMR + GPS	1505.73 (1406.7–1617.32)	2337.06 (2127.77–2584.98)	0.01 (0–0.04)	0.04 (0.02–0.08)				
gSMR	982.6 (578.57–5263.31)	1488.66 (874.65–5555.84)	0.04 (0.01–0.25)	0.11 (0.04–1.12)	0.45 (0.06–3.11)			
gSMR + GPS	1501.82 (1403.74–1606.86)	2329.8 (2118.72–2579.92)	0.02 (0–0.04)	0.04 (0.02–0.09)	0.11 (0.03–0.3)			
SCR	1485.62 (1213.18–1913.99)	2065.89 (1462.97–3516.24)				-1.24 (-1.93–-0.55)	0.46 (0.19–0.74)	-1.77 (-2.97–-0.65)
SCR + GPS	1507.05 (1411.9–1615.81)	2356.35 (2147.23–2596.73)				-1.26 (-1.81–-0.76)	0.46 (0.21–0.73)	-1.96 (-2.8–-1.16)
gSMR + SCR	1510.3 (1240.06–1937.53)	1650.86 (1305.83–2232.81)	0.01 (0–0.03)	0.03 (0.02–0.05)	0.13 (0.04–0.38)	-1.38 (-2.03–-0.75)	0.49 (0.22–0.8)	-0.98 (-1.9–-0.05)
gSMR + SCR + GPS	1505.35 (1415.79–1608.89)	2311.92 (2117.05–2533.6)	0.01 (0–0.03)	0.02 (0.01–0.04)	0.08 (0.02–0.21)	-1.37 (-1.89–-0.86)	0.47 (0.22–0.75)	-1.52 (-2.27–-0.79)

Table S5: Sex-specific density estimates for cougars across a suite of models. Densities are presented as the number of animals per 100 km^2. HPDI = highest posterior density interval. BCI = Bayesian Credible Interval. CV = coefficient of variation (defined as the standard deviation divided by the posterior mean).

**Model**	**Mean**	**Median**	**Mode**	**SD**	**Lower 95% HPDI**	**Upper 95% HPDI**	**Lower 95% BCI**	**Upper 95% BCI**	**CV**
SMR	1.05	0.7	0.2	0.97	0.12	3.08	0.16	3.59	0.92
SMR + GPS	2.25	2.15	1.91	0.83	0.82	3.86	0.94	4.1	0.37
gSMR	2.32	2.19	1.86	0.92	0.82	4.18	0.94	4.53	0.4
gSMR + GPS	2.19	2.07	1.95	0.76	0.94	3.79	1.01	3.98	0.35
SCR	1.48	1.32	0.97	0.83	0.23	3.08	0.4	3.61	0.56
SCR + GPS	2.51	2.25	1.78	1.18	0.76	4.97	0.89	5.33	0.47
SCR + gSMR	2.52	2.42	2.19	0.87	0.96	4.2	1.19	4.6	0.35
SCR + gSMR + GPS	2.39	2.28	2.16	0.69	1.22	3.81	1.29	3.97	0.29

Table S6: Detection parameters for sex-specific models estimating the densities of cougars. Values are presented as the median of the posterior distribution with 95% Bayesian Credible Intervals in parentheses. σ = spatial scale parameter, $\lambda0_{resight}$ = baseline detection rate for camera analyses, $\lambda0_{marking}$ = baseline detection rate for the marking process, $p0$ (intercept) = baseline detection probability on the logit scale for genetic SCR models when all covariates are at zero, $p0$ (survey effort) = effect of survey effort (distance traveled by scat detection dogs) on baseline detection probability in genetic SCR models on the logit scale, and $p0$(sex) = effect of being male (compared to females) on baseline detection probability in genetic SCR models on the logit scale.

Model	σ female	σ male	$\lambda0_{resight}$ female	$\lambda0_{resight}$ male	$\lambda0_{marking}$	$p0$ (intercept)	$p0$ (survey effort)	$p0$ (sex)
SMR	6388.18 (2530.12–15281.61)	5377.48 (2182.98–18565.03)	0.07 (0.01–3.6)	0.04 (0.01–0.26)				
SMR + GPS	3135.1 (2932.13–3365.81)	4799.03 (4533.41–5087.11)	0.03 (0.02–0.06)	0.02 (0.01–0.04)				
gSMR	2901.96 (2007.23–6257.69)	4407.54 (2442.08–9075.38)	0.04 (0.02–0.1)	0.03 (0.01–0.08)	0.48 (0.17–1.28)			
gSMR + GPS	3250.23 (3042.22–3482.17)	4891.93 (4635.82–5192.51)	0.03 (0.02–0.06)	0.02 (0.01–0.04)	0.43 (0.16–1.07)			
SCR	1175 (667.18–15266.91)	5164.32 (2622.39–8976.27)				-4.82 (-10.28–-0.27)	10.87 (0.45–24.71)	6.72 (0.12–15.93)
SCR + GPS	3152.54 (2947.77–3390.86)	4811.07 (4556.17–5080.18)				-4.22 (-5.98–-2.76)	0.57 (0.08–1.06)	0.81 (-1.02–2.66)
gSMR + SCR	2835.95 (2090.65–5259.9)	4596.02 (3020.71–7868.03)	0.04 (0.02–0.08)	0.03 (0.01–0.09)	0.44 (0.16–1.1)	-4.21 (-5.79–-2.96)	0.63 (0.1–1.3)	1.36 (-0.45–3.55)
gSMR + SCR + GPS	3251.37 (3045.38–3489.19)	4889.69 (4621.46–5174.91)	0.03 (0.02–0.06)	0.02 (0.01–0.04)	0.41 (0.16–0.95)	-4.32 (-5.72–-3.18)	0.58 (0.06–1.1)	1.21 (-0.38–2.92)

Table S7: Sex-specific density estimates for coyotes across a suite of models. Densities are presented as the number of animals per 100 km^2. HPDI = highest posterior density interval. BCI=Bayesian Credible Interval. CV=coefficient of variation (defined as the standard deviation divided by the posterior mean).

Model	Mean	Median	Mode	SD	Lower 95% HPDI	Upper 95% HPDI	Lower 95% BCI	Upper 95% BCI	CV
SMR	48.68	48.22	47.94	7.73	34.18	64.24	34.79	65.15	0.16
SMR + GPS	34.31	33.87	33.5	5.56	24.11	45.77	24.87	47	0.16
gSMR	43.01	42.72	42.44	6.7	30.67	56.91	30.97	57.37	0.16
gSMR + GPS	31.24	30.82	30.56	4.65	21.97	39.98	23.04	41.5	0.15
SCR	33.51	33.24	32.93	3.46	27.31	40.55	27.44	40.83	0.1
SCR + GPS	24.67	24.55	24.33	2.42	20.41	29.66	20.41	29.66	0.1
SCR + gSMR	37.58	37.38	37.09	3.65	30.07	44.55	30.76	45.24	0.1
SCR + gSMR + GPS	28.12	28	27.79	2.54	23.59	33.38	23.45	33.38	0.09

Table S8: Detection parameters for sex-specific models estimating the densities of coyotes. Values are presented as the median of the posterior distribution with 95% Bayesian Credible Intervals in parentheses. σ = spatial scale parameter, $\lambda0_{resight}$ = baseline detection rate for camera analyses, $\lambda0_{marking}$ = baseline detection rate for the marking process, $p0$ (intercept) = baseline detection probability on the logit scale for genetic SCR models when all covariates are at zero, $p0$ (survey effort) = effect of survey effort (distance traveled by scat detection dogs) on baseline detection probability in genetic SCR models on the logit scale, and $p0$(sex) = effect of being male (compared to females) on baseline detection probability in genetic SCR models on the logit scale.

Model	σ female	σ male	$\lambda0_{resight}$ female	$\lambda0_{resight}$ male	$\lambda0_{marking}$	$p0$ (intercept)	$p0$ (survey effort)	$p0$ (sex)
SMR	439.7 (385.31–497.28)	937.69 (792.48–1134.37)	1.07 (0.78–1.41)	0.22 (0.14–0.35)				
SMR + GPS	3225.55 (3011.37–3460.18)	2436.67 (2351.52–2526.31)	0.02 (0.01–0.04)	0.06 (0.04–0.08)				
gSMR	516.67 (465.44–582.56)	1090.28 (943.15–1292.26)	0.9 (0.66–1.19)	0.18 (0.12–0.27)	0.18 (0.08–0.36)			
gSMR + GPS	3220.71 (3021.79–3435.5)	2460.63 (2371.67–2549.16)	0.02 (0.01–0.04)	0.06 (0.05–0.08)	0.02 (0.01–0.04)			
SCR	1175.68 (1006.23–1418.81)	1381.25 (1184.39–1629.71)				-1.24 (-1.82–-0.67)	0.61 (0.37–0.94)	-0.35 (-1.06–0.39)
SCR + GPS	3130.83 (2933.49–3344.39)	2433.62 (2347.76–2525.48)				-2.96 (-3.39–-2.56)	0.4 (0.24–0.55)	0.39 (-0.13–0.92)
gSMR + SCR	1016.22 (841.6–1240.43)	1258.42 (1103.38–1427.17)	0.23 (0.06–0.42)	0.16 (0.09–0.29)	0.07 (0.04–0.14)	-1.06 (-1.64–-0.46)	0.62 (0.37–0.94)	-0.33 (-1.06–0.31)
gSMR + SCR + GPS	3069.76 (2871.63–3277.89)	2422.85 (2340.07–2507.23)	0.03 (0.01–0.05)	0.07 (0.05–0.09)	0.02 (0.01–0.03)	-3 (-3.37–-2.63)	0.39 (0.23–0.53)	0.29 (-0.19–0.79)
