# Supplementary material for: Evaluating and integrating spatial capture–recapture models with data of variable individual identifiability
Source: Ecol Appl. 2021 Aug 11;31(7):e02405. doi: 10.1002/eap.2405 (PMC9286611; doi:10.1002/eap.2405)
Supplement: Supplementary file 7 — Appendix S7 [file EAP-31-0-s006.pdf]

Supporting Information. Ruprecht, J.S., C.E. Eriksson, T.D. Forrester, D.A. Clark, M.J. Wisdom, M.M. Rowland, B.K. Johnson, and T. Levi. 2021. Evaluating and integrating spatial capture–recapture models with data of variable individual identifiability. Ecological Applications.

Appendix S7: Tables including measures of central tendency, detection parameters, credible intervals, and coefficients of variation for reduced sampling period density estimation models for black bears, bobcats, cougars, and coyotes. The results presented here are for analyses using cameras with a sampling period reduced from 140 days to 70 days. Models include spatial count (SC), spatial mark-resight (SMR), generalized spatial mark-resight (gSMR), spatial capture-recapture (SCR), and a novel hybrid model combining SCR and gSMR. Each model is fit with and without global positioning system (GPS) collar data from a subset of individuals within each population. See Appendix S4 for similar tables for base models, Appendix S6 for sex-specific models, and Appendix S8 for models incorporating behavioral status of coyotes.

Table S1: Density estimates for reduced sampling period models for black bears across a suite of models. Densities are presented as the number of animals per 100 km^2. HPDI = highest posterior density interval. BCI = Bayesian Credible Interval. CV = coefficient of variation (defined as the standard deviation divided by the posterior mean).

**Model**	**Mean**	**Median**	**Mode**	**SD**	**Lower 95% HPDI**	**Upper 95% HPDI**	**Lower 95% BCI**	**Upper 95% BCI**	**CV**
SC	211.97	176.22	88.95	133.19	33.72	473.19	48.95	506.92	0.63
SC + GPS	1.46	0.74	0.39	2.14	0.04	5.58	0.2	9.41	1.47
SPA	219.33	189.28	80.12	142.89	17.95	482.44	32.09	505.83	0.65
SPA + GPS	1.42	0.86	0.47	1.49	0.08	5.07	0.2	6.24	1.05
SMR	7.55	7.53	7.39	3.03	2.65	13.5	2.15	13.15	0.4
SMR + GPS	14.08	13.66	13.22	3.65	7.1	21.66	8.19	23.02	0.26
gSMR	8.37	8.39	8.44	2.85	3.39	13.62	2.73	13.27	0.34
gSMR + GPS	11.81	11.51	10.99	2.82	6.63	17.33	7.1	17.99	0.24
SCR + gSMR	12.25	12.13	11.93	2.85	7.12	18.14	7.01	18.06	0.23
SCR + gSMR + GPS	8.94	8.82	8.57	1.72	5.89	12.51	6.08	12.82	0.19

Table S2: Detection parameters for density models with a reduced sampling period for black bears. Values are presented as the median of the posterior distribution with 95% Bayesian Credible Intervals in parentheses. σ = spatial scale parameter, $\lambda0_{resight}$ = baseline detection rate for camera analyses, $\lambda0_{marking}$ = baseline detection rate for the marking process, $p0$ (intercept) = baseline detection probability on the logit scale for genetic SCR models when all covariates are at zero, $p0$ (survey effort) = effect of survey effort (distance traveled by scat detection dogs) on baseline detection rate in genetic SCR models on the logit scale.

Model	σ	$\lambda0_{resight}$	$\lambda0_{marking}$	$p0$ (intercept)	$p0$ (survey effort)
SC	199.51 (105.95–377.74)	0.69 (0.41–1.18)			
SC + GPS	6486.45 (6218.57–6777.43)	0.21 (0.01–1.44)			
SPA	288.86 (140.9-826.71)	0.23 (0.06–0.44)			
SPA + GPS	6490.86 (6219.73–6785.74)	0.12 (0.01–0.67)			
SMR	4515.35 (3082.51–8400.48)	0.03 (0.02–0.07)			
SMR + GPS	3393.82 (3247.93–3543.98)	0.03 (0.02–0.05)			
gSMR	3826.74 (2776.88–6261.02)	0.04 (0.02–0.08)	0.07 (0.03–0.19)		
gSMR + GPS	3394.4 (3249.29–3546.78)	0.04 (0.02–0.05)	0.07 (0.03–0.14)		
gSMR + SCR	3955.49 (3050.38–5531.83)	0.02 (0.02–0.04)	0.04 (0.02–0.09)	-4.34 (-4.9–-3.84)	0.41 (0.15–0.66)
gSMR + SCR + GPS	5283.01 (5060.83–5519.62)	0.02 (0.01–0.03)	0.03 (0.01–0.07)	-4.62 (-5.1–-4.18)	0.41 (0.14–0.65)

Table S3: Density estimates for reduced sampling period models for bobcats across a suite of models. Densities are presented as the number of animals per 100 km^2. HPDI = highest posterior density interval. BCI = Bayesian Credible Interval. CV = coefficient of variation (defined as the standard deviation divided by the posterior mean).

Model	Mean	Median	Mode	SD	Lower 95% HPDI	Upper 95% HPDI	Lower 95% BCI	Upper 95% BCI	CV
SC	93.35	84.14	24.51	69.5	0.47	217.77	0.94	226.74	0.74
SC + GPS	49.13	36.29	10.79	39.62	1.89	126.8	3.3	133.4	0.81
SPA	97.77	91.8	41.68	68.51	0.29	214.58	0.58	222.4	0.7
SPA + GPS	48	31.11	9.66	44.47	1.41	146.12	3.3	156.49	0.93
SMR	7.16	6.22	4.82	4.68	0.33	16.22	0.82	18.35	0.65
SMR + GPS	8.11	7.37	6.49	4.03	1.97	16.22	2.62	18.84	0.5
gSMR	5.71	5.16	4.36	3.66	0.49	12.69	0.82	14.74	0.64
gSMR + GPS	7.46	6.88	6.12	3.08	2.29	13.43	2.95	14.91	0.41
SCR + gSMR	12.43	12.29	12.18	2.04	8.52	16.46	8.85	16.95	0.16
SCR + gSMR + GPS	12.06	11.88	11.6	2.03	8.19	15.97	8.52	16.38	0.17

Table S4: Detection parameters for density models with a reduced sampling period for bobcats. Values are presented as the median of the posterior distribution with 95% Bayesian Credible Intervals in parentheses. σ = spatial scale parameter, $\lambda 0_{resight}$ = baseline detection rate for camera analyses, $\lambda 0_{marking}$ = baseline detection rate for the marking process, $p0$ (intercept) = baseline detection probability on the logit scale for genetic SCR models when all covariates are at zero, $p0$ (survey effort) = effect of survey effort (distance traveled by scat detection dogs) on baseline detection rate in genetic SCR models on the logit scale.

Model	σ	$\lambda 0_{resight}$	$\lambda 0_{marking}$	$p0$ (intercept)	$p0$ (survey effort)
SC	475.33 (106.76–17124.04)	0.03 (0–0.31)			
SC + GPS	1829.84 (1730.92–1941.85)	0 (0–0.04)			
SPA	422.74 (127.9–17498.85)	0.04 (0–0.31)			
SPA + GPS	1829.34 (1726.29–1935.73)	0.01 (0–0.07)			
SMR	1786.87 (966.1–6181.45)	0.04 (0.01–0.1)			
SMR + GPS	1833.82 (1735.24–1940.59)	0.03 (0.01–0.06)			
gSMR	1871.11 (1066.05–4825.41)	0.04 (0.01–0.14)	0.23 (0.04–1.25)		
gSMR + GPS	1826.77 (1734.3–1933.84)	0.03 (0.01–0.06)	0.12 (0.03–0.31)		
gSMR + SCR	1654.18 (1405.55–2005.92)	0.02 (0.01–0.04)	0.12 (0.03–0.31)	-1.98 (-2.45–-1.52)	0.45 (0.2–0.75)
gSMR + SCR + GPS	1825.5 (1732.81–1932.5)	0.02 (0.01–0.03)	0.08 (0.02–0.2)	-2.16 (-2.55–-1.78)	0.45 (0.2–0.73)

Table S5: Density estimates for reduced sampling period models for cougars across a suite of models. Densities are presented as the number of animals per 100 km^2. HPDI = highest posterior density interval. BCI = Bayesian Credible Interval. CV = coefficient of variation (defined as the standard deviation divided by the posterior mean).

Model	Mean	Median	Mode	SD	Lower 95% HPDI	Upper 95% HPDI	Lower 95% BCI	Upper 95% BCI	CV
SC	156.94	141.23	92.3	100.39	1.46	336.68	9.1	351.24	0.64
SC + GPS	5.44	3.82	0.93	4.85	0.04	15.8	0.35	17.52	0.89
SPA	25.31	16.78	1.81	24.05	0.08	71.87	0.25	75.96	0.95
SPA + GPS	5.63	3.98	0.92	4.89	0.12	15.8	0.35	17.48	0.87
SMR	2	1.76	0.25	1.54	0.12	4.96	0.16	5.7	0.77
SMR + GPS	2.02	1.87	1.72	0.83	0.7	3.63	0.78	3.98	0.41
gSMR	2.44	2.26	1.94	1.08	0.55	4.45	0.9	5.07	0.44
gSMR + GPS	1.7	1.6	1.49	0.6	0.66	2.89	0.78	3.12	0.35
SCR + gSMR	2.35	2.22	1.97	0.8	0.99	3.94	1.13	4.17	0.34
SCR + gSMR + GPS	1.88	1.82	1.65	0.54	0.93	2.95	1.03	3.08	0.29

Table S6: Detection parameters for density models with a reduced sampling period for cougars. Values are presented as the median of the posterior distribution with 95% Bayesian Credible Intervals in parentheses. σ = spatial scale parameter, $\lambda 0_{resight}$ = baseline detection rate for camera analyses, $\lambda 0_{marking}$ = baseline detection rate for the marking process, $p0$ (intercept) = baseline detection probability on the logit scale for genetic SCR models when all covariates are at zero, $p0$ (survey effort) = effect of survey effort (distance traveled by scat detection dogs) on baseline detection rate in genetic SCR models on the logit scale.

Model	σ	$\lambda 0_{resight}$	$\lambda 0_{marking}$	$p0$ (intercept)	$p0$ (survey effort)
SC	254.91 (106.39–1671.55)	0.1 (0.01–0.36)			
SC + GPS	5068.05 (4877.84–5282.71)	0.01 (0–0.11)			
SPA	2818.17 (349.01–18561.7)	0.01 (0–0.12)			
SPA + GPS	5066.44 (4867.55–5268.41)	0.01 (0–0.09)			
SMR	4365.04 (2216.4–18414.61)	0.03 (0.01–0.07)			
SMR + GPS	5055.87 (4862.87–5255.02)	0.02 (0.01–0.03)			
gSMR	3547.05 (2392.2–6451.37)	0.03 (0.01–0.06)	0.39 (0.13–1.19)		
gSMR + GPS	5196.2 (5002.76–5410.99)	0.02 (0.01–0.03)	0.32 (0.11–0.81)		
gSMR + SCR	3630.5 (2658.02–5739.51)	0.03 (0.02–0.06)	0.4 (0.15–1.06)	-3.53 (-4.44–-2.69)	0.54 (0.04–1.04)
gSMR + SCR + GPS	4432.37 (4258.04–4611.01)	0.03 (0.02–0.04)	0.43 (0.15–1.02)	-3.69 (-4.48–-3.02)	0.53 (0.07–1.02)

Table S7: Density estimates for reduced sampling period models for coyotes across a suite of models. Densities are presented as the number of animals per 100 km^2. HPDI = highest posterior density interval. BCI = Bayesian Credible Interval. CV = coefficient of variation (defined as the standard deviation divided by the posterior mean).

**Model**	**Mean**	**Median**	**Mode**	**SD**	**Lower 95% HPDI**	**Upper 95% HPDI**	**Lower 95% BCI**	**Upper 95% BCI**	**CV**
SC	125.15	115.01	95.53	50.32	50.91	223.43	52.32	226.73	0.4
SC + GPS	3.42	2.75	1.86	2.49	0.61	7.32	1.07	9.61	0.73
SPA	108.44	97.1	82.67	49.95	33.47	214	38.65	222.02	0.46
SPA + GPS	4.7	3.77	2.37	2.92	1.89	10.37	1.89	12.26	0.62
SMR	35.85	35.32	34.82	6.72	22.12	48.22	24.11	50.66	0.19
SMR + GPS	22.69	22.28	21.77	4.38	14.5	31.28	15.11	32.35	0.19
gSMR	36.55	35.7	34.74	7.26	24.11	51.42	24.41	52.34	0.2
gSMR + GPS	25.48	25.02	24.5	4.47	17.24	34.48	17.85	35.4	0.18
SCR	33.96	33.79	33.47	3.26	27.86	40.28	28.28	40.69	0.1
SCR + GPS	25.51	25.38	25.2	2.24	21.24	29.93	21.38	30.34	0.09
SCR + gSMR	125.15	115.01	95.53	50.32	50.91	223.43	52.32	226.73	0.4
SCR + gSMR + GPS	3.42	2.75	1.86	2.49	0.61	7.32	1.07	9.61	0.73

Table S8: Detection parameters for density models with a reduced sampling period for coyotes. Values are presented as the median of the posterior distribution with 95% Bayesian Credible Intervals in parentheses. σ = spatial scale parameter, $\lambda 0_{resight}$ = baseline detection rate for camera analyses, $\lambda 0_{marking}$ = baseline detection rate for the marking process, $p0$ (intercept) = baseline detection probability on the logit scale for genetic SCR models when all covariates are at zero, $p0$ (survey effort) = effect of survey effort (distance traveled by scat detection dogs) on baseline detection rate in genetic SCR models on the logit scale.

**Model**	**σ**	$\boldsymbol{\lambda 0_{resight}}$	$\boldsymbol{\lambda 0_{marking}}$	$\boldsymbol{p0}$ **(intercept)**	$\boldsymbol{p0}$ **(survey effort)**
SC	278.19 (181.29–416.37)	0.99 (0.67–1.44)			
SC + GPS	2671.04 (2585.92–2760.22)	0.6 (0.16–1.18)			
SPA	384.45 (225.17–642.21)	0.44 (0.24–0.66)			
SPA + GPS	2729.21 (2644.5–2818.39)	0.29 (0.09–0.61)			
SMR	656.43 (578.58–759.43)	0.62 (0.42–0.9)			
SMR + GPS	2639.39 (2560.32–2729.72)	0.06 (0.04–0.08)			
gSMR	849 (730.45–1018.26)	0.36 (0.24–0.53)	0.15 (0.06–0.31)		
gSMR + GPS	2656.85 (2574.93–2742.68)	0.05 (0.04–0.07)	0.02 (0.01–0.04)		
gSMR + SCR	1272.99 (1158.08–1408.9)	0.16 (0.12–0.2)	0.07 (0.03–0.12)	-1.41 (-1.73–-1.1)	0.6 (0.38–0.89)
gSMR + SCR + GPS	2595.27 (2511.87–2677.82)	0.05 (0.04–0.06)	0.02 (0.01–0.04)	-2.71 (-2.94–-2.48)	0.38 (0.23–0.53)
