# Supplementary material for: Evaluating and integrating spatial capture–recapture models with data of variable individual identifiability
Source: Ecol Appl. 2021 Aug 11;31(7):e02405. doi: 10.1002/eap.2405 (PMC9286611; doi:10.1002/eap.2405)
Supplement: Supplementary file 9 — Data S1 [file EAP-31-0-s003.zip › DataS1/MetadataS1.pdf]

**Ruprecht, J.S., C.E. Eriksson, T.D. Forrester, D.A. Clark, M.J. Wisdom, M.M. Rowland, B.K. Johnson, and T. Levi. 2021. Evaluating and integrating spatial capture–recapture models with data of variable individual identifiability. Ecological Applications.**

---

## Data S1

**Data and R code to fit spatial density estimation models for black bears, bobcats, cougars and coyotes.**

---

## Authors of the material provided in DataS1.zip


Joel S. Ruprecht
Oregon State University
104 Nash Hall, Corvallis, OR 97331
joel.ruprecht@oregonstate.edu

Charlotte E. Eriksson
Oregon State University
104 Nash Hall, Corvallis, OR 97331
charlotte.eriksson@oregonstate.edu

Tavis D. Forrester
Oregon Department of Fish and Wildlife
1401 Gekeler Lane, La Grande, OR 97850
tavis.d.forrester@state.or.us

Darren A. Clark
Oregon Department of Fish and Wildlife
1401 Gekeler Lane, La Grande, OR 97850
darren.a.clark@state.or.us

Michael J. Wisdom
Pacific Northwest Research Station, USDA Forest Service
1401 Gekeler Lane, La Grande, OR 97850
michael.wisdom@usda.gov

Mary M. Rowland
Pacific Northwest Research Station, USDA Forest Service
1401 Gekeler Lane, La Grande, OR 97850
mary.rowland@usda.gov

Bruce K. Johnson
Oregon Department of Fish and Wildlife
1401 Gekeler Lane, La Grande, OR 97850
rowljohn@gmail.com

Taal Levi
Oregon State University
104 Nash Hall, Corvallis, OR 97331
taal.levi@oregonstate.edu

---

## File list (files found within DataS1.zip)

```
coyoteData.RData
cougarData.RData
bearData.RData
bobcatData.RData
JAGS_NIMBLE_code_all_models.txt
run_hybrid_coyote_nimble_parallel.R
```

## Description

`coyoteData.RData` – data to fit spatial density models for coyotes.
`cougarData.RData` – data to fit spatial density models for cougars.
`bearData.RData` – data to fit spatial density models for black bears.
`bobcatData.RData` – data to fit spatial density models for bobcats.
`JAGS_NIMBLE_code_all_models.txt` – JAGS/NIMBLE code to fit spatial presence absence, spatial count, conventional spatial mark-resight, generalized spatial mark-resight, spatial capture-recapture, and hybrid generalized spatial mark-resight models incorporating GPS telemetry data.
`run_hybrid_coyote_nimble_parallel.R` – program to fit the hybrid gSMR+SCR with telemetry model for coyotes in NIMBLE using parallel processing.

---
